# Modeling the Effects of Calcium Overload on Mitochondrial Ultrastructural Remodeling

**DOI:** 10.3390/app11052071

**Published:** 2021-02-26

**Authors:** Jasiel O. Strubbe-Rivera, Jiahui Chen, Benjamin A. West, Kristin N. Parent, Guo-Wei Wei, Jason N. Bazil

**Affiliations:** 1 Department of Pharmacology and Toxicology, Michigan State University, East Lansing, MI 48824, USA; 2 Department of Mathematics, Michigan State University, East Lansing, MI 48824, USA; 3 Department of Physiology, Michigan State University, East Lansing, MI 48824, USA; 4 Department of Biochemistry and Molecular Biology, Michigan State University, East Lansing, MI 48824, USA; 5 Department of Electrical and Computer Engineering, Michigan State University, East Lansing, MI 48824, USA

**Keywords:** bioenergetics, calcium overload, computer simulation, cristae, cryo-electron microscopy, mathematical modeling, membrane topology, mitochondria, mitochondrial dynamics, mitochondria ultrastructure

## Abstract

Mitochondrial cristae are dynamic invaginations of the inner membrane and play a key role in its metabolic capacity to produce ATP. Structural alterations caused by either genetic abnormalities or detrimental environmental factors impede mitochondrial metabolic fluxes and lead to a decrease in their ability to meet metabolic energy requirements. While some of the key proteins associated with mitochondrial cristae are known, very little is known about how the inner membrane dynamics are involved in energy metabolism. In this study, we present a computational strategy to understand how cristae are formed using a phase-based separation approach of both the inner membrane space and matrix space, which are explicitly modeled using the Cahn–Hilliard equation. We show that cristae are formed as a consequence of minimizing an energy function associated with phase interactions which are subject to geometric boundary constraints. We then extended the model to explore how the presence of calcium phosphate granules, entities that form in calcium overload conditions, exert a devastating inner membrane remodeling response that reduces the capacity for mitochondria to produce ATP. This modeling approach can be extended to include arbitrary geometrical constraints, the spatial heterogeneity of enzymes, and electrostatic effects to mechanize the impact of ultrastructural changes on energy metabolism.

## 1. Introduction

Mitochondria are small organelles that provide the bulk of the energy requirements for nearly all cells in most eukaryotic organisms. They convert chemical energy in foodstuffs into a chemical potential called the ATP hydrolysis potential. Cells use this potential to synthesize proteins, power mechanical motion, sustain homeostasis, and perform other life-supporting functions. In the heart, the essential importance of mitochondria is evidenced by the fact that they constitute up to 30% of the total cardiomyocyte volume. For example, in the absence of functional mitochondria, the heart will exhaust all of its ATP reserves in less than a minute [1], so even brief interruptions in mitochondrial ATP production have devastating consequences on energy homeostasis. Such interruptions in ATP production are caused by a myriad of diseases and conditions, with calcium overload being one of the more prominent causes. While low matrix-free calcium (<5 μM) activates matrix dehydrogenases and improves oxidative phosphorylation [2-4], calcium overload decreases ATP synthesis in a variety of ways [5-22]. In extreme cases of calcium overload, there is a complete loss of mitochondrial ATP production caused by mitochondrial rupture and fragmentation [23-25]. The precise mechanisms responsible for these effects are unknown but appear to be associated with ultrastructural changes caused by calcium overload [14,25].

Mitochondria consist of two compartments formed by two separate membranes: the outer membrane and the inner membrane. The outer membrane encases the inner membrane and serves as the gateway between the cytosolic and mitochondrial environments. This membrane forms transient connections with extra-mitochondrial organelles, such as the endoplasmic reticulum, ribosomes, nucleus, and inner membrane. The intermembrane space (IMS) compartment is between the outer and inner membrane, and the matrix compartment is encapsulated by the inner membrane. The inner membrane surface area is approximately five to ten times that of the outer membrane and forms compartments called cristae that fit within the envelope of the outer membrane. Cristae are invaginations of the inner membrane that contain the bulk of the metabolic enzymes required to produce and deliver ATP to the cell. Both membranes are under the active control of fission and fusion proteins that react to external stimuli and energy demand cues to coordinate ATP production [26-28]. While the major fission and fusion proteins of the outer membrane are known, much less is known about the key proteins involved in inner membrane dynamics. Even less is known about regulation of the inner membrane structures, such as cristae and cristae junctions.

While we still lack a complete picture of cristae formation and how they are regulated, some of the key proteins involved have been identified [29]. Cristae are not static structures [30]; they are dynamically formed in a manner dependent on a variety of known and unknown factors which control ATP synthesis rates [31]. That said, the main proteins that exert some control over the cristae’s shape are the mitochondrial contact site and cristae organizing system (MICOS) [32-34], optic atrophy factor 1 (OPA1) [33,35,36], and ATP synthase [32,37-39]. The MICOS proteins are necessary to form proper cristae junctions, a region in mitochondria that connects the inner boundary membrane to the cristae lumen. OPA1 is a protein with several isoforms and proteolytic variants that serves multiple roles [40]. These proteins coordinate with the MICOS proteins to form the cristae junction and help shape the cristae’s morphology. ATP synthase dimers contribute to cristae morphology by inducing inner membrane curvature. Cardiolipin also plays an important role in regulating the cristae shape and membrane curvature [41]. A layer of regulation above this includes proteases and chaperones that process these structural proteins, such as OMA1 [42], YME1L [42], and prohibitins [43,44], which are also intricately involved in a signaling network that controls cristae morphology. However, the precise biophysical mechanisms that govern the shape of the inner membrane, including inner membrane fission and cristae structure, remain elusive.

A few modeling studies have explored the potential role of mitochondrial proteins in the inner membrane architecture and metabolic function [45-54]. These studies investigate a range of phenomena, including how proteins and effectors influence inner membrane curvature and intramatrix diffusion anomalies. However, none have looked at the dynamics of the IMS and matrix volumes under healthy and diseased conditions. In addition, there are no computational studies focusing on how the inner membrane morphology influences ATP synthesis rates. Therefore, we propose a novel computational modeling approach capable of predicting the intermembrane and matrix space geometry under physiological and pathophysiological conditions.

The spontaneous pattern formation and evolution of a two-component system can be described by a number of models, including the Canham–Helfrich curvature functional model, the Ginzburg–Landau theory, and the Cahn–Hilliard (CH) free energy [55-58]. Among them, the Canham–Helfrich model is typically used to describe cell membrane pattern formation in a water-based environment, driven by the curvature energy per unit area of the closed lipid bilayer, osmotic pressure, and surface tension [59]. This model captures cell membrane patterns without the molecular dynamics of the lipid bilayer. The Ginzburg–Landau theory describes the phase transition of type-I superconductors, the superconducting state, and the normal state without examining their microscopic electronic and magnetic properties. In the setting of a complex vector bundle over a compact Riemannian manifold, the Ginzburg–Landau theory interconnects differential geometry, quantum field theory, Yang–Mills–Higgs equations, and string theory, among others [60,61]. The CH model is widely used in material science, chemistry, and biology to describe the phase separation of a binary mixture [62]. It is the simplest model among the aforementioned theories. The CH model can be derived from the classic conservation law and free energy minimization, with the free energy function determining the final composition of the binary system [63,64]. The pattern and morphology of the binary system is further determined by the domain size and geometric shape [65,66]. The CH model can be modified to describe the nucleation dynamics and process of a specific species in the mixture [67,68]. Additionally, multicomponent CH equations have been proposed to account for the phase separation of a system consisting of more than two species [69,70]. These variations of the CH model enable researchers to develop CH-based theories and algorithms to understand the chemistry, physics, biology, and material sciences in a complex system that is intractable with detailed microscopic descriptions.

For this study, we analyzed a set of cryo-electron tomograms of healthy and calcium-overloaded mitochondria. These data show that the mitochondrial ultrastructural morphology undergoes dramatic changes in the presence of mitochondrial calcium phosphate granules. However, it is unclear how mitochondrial calcium phosphate granules regulate the mitochondrial ultrastructure. The tomographic data, combined with the respirometry data presented herein, support the concept that such morphological changes lead to a significant reduction in the mitochondrial ATP production rate. Based on these data, we speculate that the mitochondrial ATP production rate correlates with the types and numbers of cristae and cristae junctions. Our data show that a calcium overload causes significant remodeling of the inner membrane, which likely explains the decrease in mitochondrial respiration.

To explore the dynamics of the pattern formation and phase separation of the mitochondrial ultrastructure, we used the CH model as a framework. We initially modeled the mitochondrial ultrastructure as a two-component system, consisting of the inner membrane space and the matrix space. The mitochondrial outer membrane served as the domain boundary of the system. We used a free energy function for the inner membrane and a fluid matrix to demonstrate that minimizing the free energy inside a given outer membrane generates spontaneous folding of the inner membrane and the formation of layered structures, namely cristae. Next, we modeled the mitochondrial ultrastructural remodeling caused by calcium phosphate granules by using a volume exclusion approach in the CH model. By using this phase separation-based modeling approach, we show that the presence of calcium phosphate granules exerts a devastating remodeling effect on the inner membrane, which results in matrix expansion, IMS contraction, and cristae remodeling. These morphological changes correlate well with the observed changes in the ATP production rates. Future studies will further elucidate this structure–function relationship and mechanize how the inner membrane morphology impacts mitochondrial metabolism.

## 2. Materials and Methods

### Ethical Approval.

This work conformed to the National Institutes of Health’s Guide for the Care and Use of Laboratory Animals and was approved by Michigan State University’s Institutional Animal Care and Use Committee.

### Mitochondria Isolation and Protein Quantification.

Cardiac mitochondria were isolated from guinea pig hearts using differential centrifugation, as described previously [8,14,22]. Briefly, Hartley albino guinea pigs weighing 350–450 g (4–6 weeks) were injected with heparin (500 units/mL) in the intraperitoneal cavity. The animals were anesthetized with 4–5% isoflurane and decapitated by a guillotine. After decapitation, a thoracotomy was performed, and the heart was perfused with a cold cardioplegia solution and homogenized using a handheld electronic homogenizer at 18,000 rpm for 20 s. The mitochondrial protein content was quantified using the BIO-RAD bovine serum albumin (BSA) standard set kit and the bicinchoninic acid (BCA) assay. The mitochondrial suspension was diluted to a working concentration of 40 mg/mL and kept on ice for the duration of the experiment (2–3 h). The substrate stock solutions were neutralized to a pH of 7.0.

### Mitochondrial Quality Control.

The mitochondrial quality was determined using an Oxygraph 2k (Oroboros Instruments Corp., Innsbruck, Austria). The O2k chambers were loaded with 2 mL of a respiratory buffer containing 130 mM KCl, 5 mM K_2_HPO_4_, 20 mM 3-(N-morpholino)propanesulfonic acid (MOPS), 1 mM MgCl_2_, 1 mM ethylene glycol-bis(β-aminoethyl ether)-N,N,N′,N′-tetraacetic acid (EGTA), and 0.1 % (w/v) BSA at a pH of 7.1 and 37 °C. All subsequent experiments were done using this buffer and temperature. At 0 min, 5 mM sodium pyruvate and 1 mM L-malate were added, followed by 0.1 mg/mL of mitochondria. Here, we defined the leak state as the rate of oxygen consumption by mitochondria only in the presence of substrates. At 5 min, a 500 μM bolus of adenosine diphosphate (ADP) was added to induce maximal ADP-stimulated respiration. Quality was assessed by computing the respiratory control ratio (maximal ADP-stimulated rate divided by the leak rate). Only mitochondria with a respiratory control ratio (RCR) value greater than or equal to 16 were used in the experiments.

### Calcium Effects on Respiration and Oxidative Phosphorylation.

The calcium effects on mitochondrial leaking and ADP-stimulated respiration were determined by quantifying changes in the proton leak and ADP-stimulated respiration rates after a calcium challenge. At 0 min, 5 mM sodium pyruvate, 1 mM L-malate, and 0.1 mg/mL of mitochondria were injected into each 2 mL chamber containing the respiratory buffer. At 5 min, a calcium bolus of 50 μM of calcium chloride was injected. At 10 min, 500 μM of ADP was added induce the maximal ADP-stimulated respiration.

### Cryo-Electron Microscopy (Cryo-EM) Sample Vitrification and Imaging.

Isolated mitochondria were suspended at a concentration of 0.1 mg/mL in a 2 mL respiration buffer with 5 mM sodium pyruvate and 1 mM L-malate. At the collection times indicated, 5 μL samples were pipetted from the mitochondrial suspension and deposited on Quantifoil R2/2 holey carbon grids that were pretreated for 1 min using a Pelco EasiGlo glow discharge unit. Prior to the mitochondria addition, 5–10 μL of 10 nm nanogold markers were applied to the grids, which were then air dried. These beads provided markers for fiducial alignment of the tilt series. The grids were blotted to thin the water layer and subsequently plunged into liquid ethane at room temperature using a Vitrobot Mark IV with automated temperature regulation, blotting, and humidity control (Fisher Scientific). The Vitrobot chamber was set at 4 °C with 100% humidity, and the grids were blotted with a blot force of 1. The grids were then transferred and stored in liquid nitrogen until imaging. Tilt series were performed using an FEI Talos Arctica at 200 keV in low-dose conditions, operated using Thermofisher’s Tomography software. Images were collected in linear mode on a Falcon 3EC direct electron detector with an electron dose of ~2e^−^/A^2^ per tilt image. The images were collected at 22,000× magnification (4.7 A/pixel). The total electron dose was approximately 100 e^−^/Å^2^ for each tomogram.

### Tomogram Reconstruction and Modeling.

Motion correction was performed for the individual micrographs and binned by an index factor of 6 using Motioncor2 v1.2.6. Tilt series alignment was performed using IMOD v4.9.12 and standard tomographic reconstruction particles, using the Simultaneous Iterative Reconstruction Technique (SIRT) strategy. Key features of the tomograms were traced using the drawing tools functionality in IMOD (3dmod) [71].

### Statistics.

**Figure 1.**
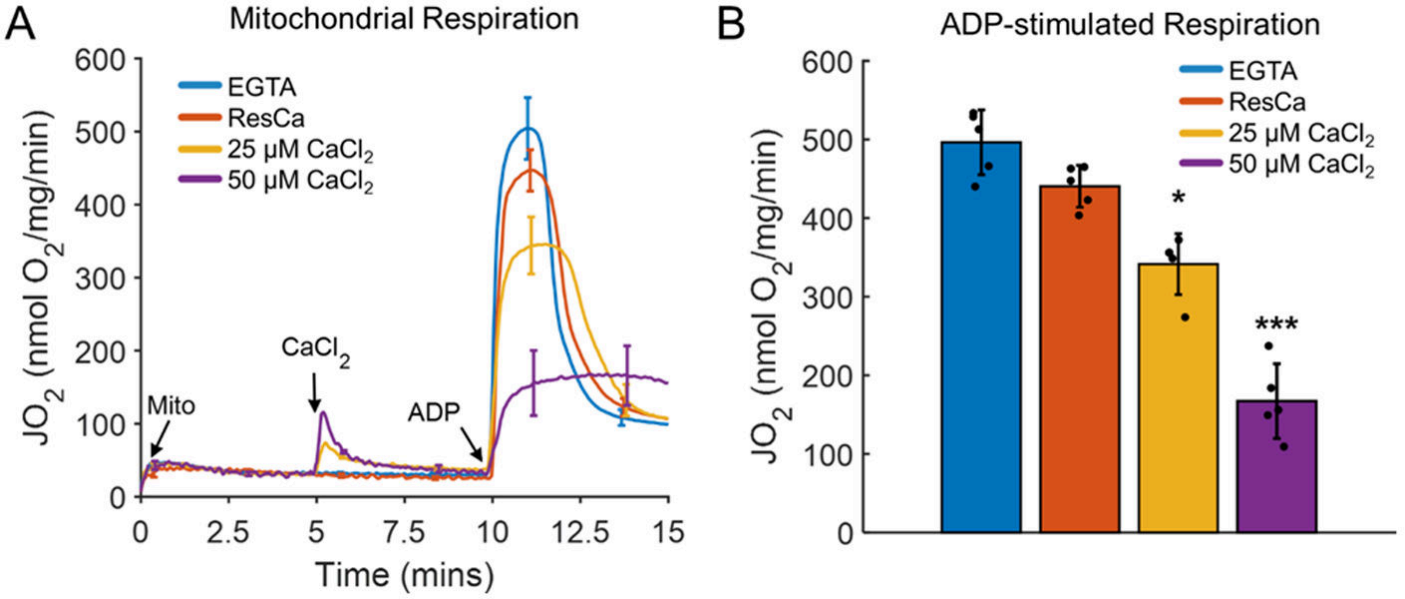
Oxidative phosphorylation decreasing proportionally with the calcium load. (**A**) Representative mitochondrial respiration profiles in the presence of the calcium chelator EGTA (~4 μM residual calcium (ResCa)) and after a 25 μM or 50 μM bolus of calcium. The mitochondrial calcium loads for these conditions were 0, 40, 250, and 500 nmol calcium/mg mitochondria, respectively. Mitochondria were energized with 5 mM sodium pyruvate and 1 mM L-malate. (**B**) The average maximum ADP-stimulated respiration rate decreased dramatically at higher calcium doses. Maximal ADP-stimulated respiration was induced following the addition of a 500 μM ADP bolus. Data are presented as the mean ± standard deviation for a sample size of five biological replicates. Here, a * and *** represent *p* values < 0.01 and <0.001, respectively.

The Shapiro–Wilks test was used to confirm data normality. All data were analyzed and plotted using MATLAB 2020a (Mathworks, Inc., Natick, MA, USA). The data in Figure 1 (n = 3–4) are presented as a mean standard deviation. An unpaired Student’s *t*-test was used to compare the calcium treatment with the control group. A value of *p* < 0.05 was assumed to be statistically significant.

### Modeling.

The dimensionless form of the Cahn–Hilliard equation is given by

(1) $$\frac{\partial \Psi }{\partial t}=\frac{1}{2}\nabla^{2}\left(-\gamma \nabla^{2}\Psi -\Psi +\Psi^{3}\right)$$

where $\sqrt{\gamma }$ is the length of the transition regions between the domain and Ψ (**r**, *t*), (Ψ (**r**, *t*) V ≤ 1) is a scalar order parameter chosen to be the difference between the local densities of the inner membrane [Ψ = −1] and the fluid matrix [Ψ = 1]. The Cahn–Hilliard equation is a fourth-order nonlinear partial differential equation (PDE), and that does not admit a general analytical solution. Numerical simulation is the major approach to obtaining the solution of the Cahn–Hilliard equation. However, some theoretical analysis can offer a basic understanding of its behavior and underlying physics. First, it is important to note that Equation (1) can be cast in its conversational form:

(2) $$\frac{\partial \Psi }{\partial t}=-\nabla \cdot J$$

where $J=-\frac{1}{2}\nabla \mu$ is a flux vector and the chemical potential of the binary system is expressed as

(3) $$\mu =\left(\Psi^{2}-1-\gamma \nabla^{2}\right)\Psi$$

This potential-driven flow has a trivial solution at Ψ = 0. Additionally, two other asymptotic solutions are given by Ψ^2^ – 1 = 0 (i.e., Ψ = ±1), which represents two phases. Since the amplitude of the leading term in Equation (3) is one, the characteristic length of the crista is one in the present setting. Therefore, the domain size should be chosen accordingly for a given set of experimental data. The boundary condition, meanwhile, is the usual zero-flux condition for the chemical potential *μ* and the scale order parameter Ψ:

(4) n⋅∇μ(r,t)=0andn⋅∇Ψ(r,t)=0,r∈Γ

where *n* is normal to the boundary and Γ is the boundary of the geometrical shape. When introducing calcium into the mitochondria system, the boundary condition around the calcium is given as

(5) $$n\cdot \nabla \mu (r,t)=0\;\text{and}\;\Psi (r,t)=C,r\in \Gamma^{\prime }$$

where *C* is a constant and Γ′ is the boundary of the region [Ψ = *C*] corresponding to the calcium region.

Moreover, the Cahn–Hilliard equation is related to the time-dependent Ginzburg–Landau equation, which can be derived from the free energy minimization. Similarly, the Cahn–Hilliard equation can also be derived from the minimization of the Ginzburg–Landau-type free energy functional *F*:

(6) $$F(\Psi )=\int_{\Omega }\left[f(\Psi )+\frac{\gamma }{2}(\nabla \Psi {)}^{2}\right]dr$$

where Ω is the domain of interest and $f(\Psi )=\frac{1}{4}{\left(\Psi^{2}-1\right)}^{2}$ is the free energy per unit of volume. It is easy to show that the Ginzburg–Landau-type free energy functional decays with respect to time:

(7) $$\frac{dF}{dt}=-\int_{\Omega }|\nabla \mu {|}^{2}dr$$

This allows us to set various initial values, such as random noise, when solving the CH equation.

Furthermore, we can linearize the CH equation to understand its long wavelength instability in spatially extended settings. Let us assume that the system is near the trivial solution Ψ(**r**, *t*) = 0, implying a uniform steady state corresponding to the homogeneous mixture. Consider the following form of solution for a small fluctuation of the order parameter:

(8) $$\Psi (r,t)=\Psi_{0}e^{\omega t}e^{ik\cdot r}$$

where **k** is the wavevector, *k* = ∣**k**∣ is the wavenumber, and *w*(*k*) is the angular frequency. The stability of the solution depends on the angular frequency. The system is linearly unstable when *w*(*k*) > 0 and decays when *w*(*k*) < 0. By substituting this solution into the linearized Cahn–Hilliard equation, we have the dispersion relation

(9) $$\omega (k)=-\frac{1}{2}\left(k^{4}-k^{2}\right)$$

Therefore, long wavelength states with *k* < 1 will be linearly unstable. Such a long wavelength instability is exploited by the nonlinear CH system. Short wavelength states with *k* > 1 will decay in a linear fashion and convert short wavelength modes (i.e., large domains) into spatiotemporal patterns. The system regulates the exponential growth tendency of long wavelength modes caused by random noise and organizes them into spatially coherent states. Therefore, the spatiotemporal patterns of certain characteristic wavelengths are stabilized in the CH equation. Due to computational challenges, most simulations were carried out in rectangular domains. Motivated by the microscopic phase separation and pattern formation of the spherical and cylindrical molecular assembly, Guan et al. considered the Cahn–Hilliard equation in a circular domain [39]. In the circular domain geometry, certain symmetric patterns occurred frequently during the simulation. These authors found that the solution of the Cahn–Hilliard equation could be effectively analyzed with Fourier–Bessel modes [40]. In the current work, the finite element method (FEM) was considered due to its advantage in dealing with irregular domains. The Cahn–Hilliard equation is a fourth-order equation whose weak form would result from the presence of second-order spatial derivatives. Solving such a form with a standard Lagrange finite element basis is problematic. Therefore, Equation (1), with the boundary condition Equation (4), is reformulated as two coupled second-order equations:

(10) $$\frac{\partial \Psi }{\partial t}-\frac{\gamma }{2}\nabla^{2}\mu =0,r\in \Omega$$

(11) $$\mu -\frac{df(\Psi )}{d\Psi }+\nabla^{2}\Psi =0,r\in \Omega$$

where the unknowns are Ψ and *μ*. Then, the variational forms of Equations (10) and (11) are

(12) $$\left(\frac{\partial \Psi }{\partial t},u\right)+\frac{\gamma }{2}a(\mu ,u)=0,\forall u\in V$$

(13) $$(\mu ,v)-\left(\frac{df(\Psi )}{d\Psi },v\right)-a(\Psi ,v)=0,\forall v\in V$$

where *u* and *ν* are test functions of the trial space *V*, (*u*, *ν*) is the *L*_2_ inner product which represents the integration of the product of *u* and *ν* over the region, and *a*(*u*, *ν*) is the bilinear form, such that *a*(*u*, *ν*) = (∇*u*, ∇*ν*). As for time discretization, the theta method is applied to the variational forms of the equation:

(14) $$\left(\frac{\Psi_{n+1}-\Psi_{n}}{\Delta t},u\right)+\frac{\gamma }{2}a(\mu_{n+\theta },u)=0,\forall u\in V$$

(15) $$(\mu_{n+1},v)-\left(\frac{df_{n+1}}{d\Psi },v\right)-a(\Psi_{n+1},v)=0,\forall v\in V$$

where Δ*t* = *t*_*n*+1_ – *t*_*n*_ and *μ*_*n*+*θ*_ = (1 – *θ*)*μ*_*n*+1_ + *θμ*_*n*_. The simulations operate on 2D triangle meshes and 3D tetrahedral meshes. In the following tests, we first present the simulations of a mitochondrial system without calcium using the CH models and then give the simulations of the system with calcium. Numerical implementation is done by applying the FEniCS [51], and simulation plots are generated by ParaView [52].

## 3. Results and Discussion

Oxidative phosphorylation was inhibited in the calcium overload state [5-22]. As the calcium load increased, the maximal ADP-stimulated respiration was significantly inhibited, as shown in Figure 1. This phenomenon is not associated with the well-known mitochondrial permeability transition phenomenon [8]. At these calcium loads, the mitochondrial calcium, membrane potential, and respiration were stable [8,14]. The inhibitory effect began at calcium loads of around 50 nmol/mg of mitochondrial protein. This value was calculated based on the quantified calcium uptake dynamics of the residual calcium in one of our prior studies [22]. This inhibition got progressively worse as the calcium load increased up to 500 nmol/mg. Therefore, calcium loads in the range of 50–500 nmol/mg maximally stimulated the ADP-respiration rates to decrease in a titratable manner. The cause of this inhibition was suspected to originate from calcium phosphate granule-induced cristae remodeling in an earlier study [14]. Below this range, calcium is beneficial for mitochondrial function as it stimulates matrix dehydrogenases [4]. Above this range, mitochondria undergo mitochondrial permeability transition, deenergize, and become net ATP consumers. Specifically, the mitochondrial inner membrane becomes permeable to solutes in the low kDa range, effectively preventing their ability to store chemical energy in the form of a proton electrochemical gradient. Decades of research has been done on this phenomenon [72-75], but little is known about how mitochondria respond to calcium loads below the level which induces this phenomenon and above those used for energy homeostasis. Therefore, we explored how calcium loads that do not cause permeability transition but inhibit oxidative phosphorylation alter the mitochondrial ultrastructure.

**Figure 2.**
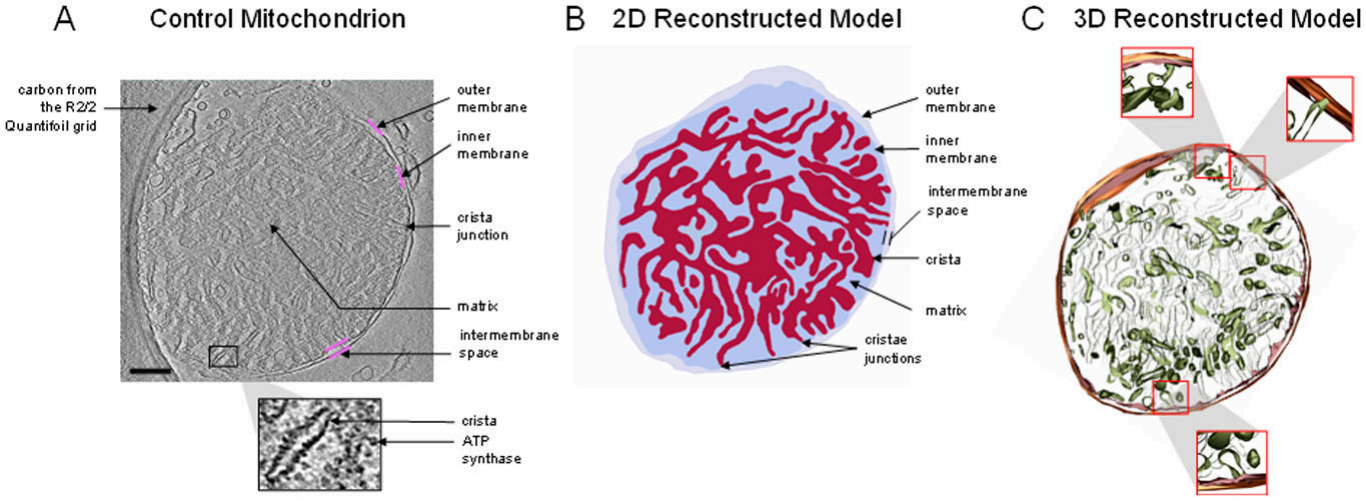
Mitochondrion with an abundant, interconnected cristae. (**A**) Representative portion of the tomogram from a mitochondrion in the absence of calcium. The sample was taken approximately five minutes after mitochondria were added to the respiration buffer. To remove all calcium from the system, 1 mM EGTA was added before the addition of the mitochondria. The image resolution is approximately 4 Å per pixel. The solid magenta lines highlight inner or outer membrane boundaries. Structures of interest, including the ATP synthase, crista, and crista junctions, are labeled. (**B**) The reconstructed model shows a complex cristae distribution, occupying nearly half of the total volume. The small, black, dot-like structures are ATP synthase molecules. (**C**) The 3D reconstructed model shows a densely packed, interconnected cristae network. Scale bar = 250 nm.

**Figure 3.**
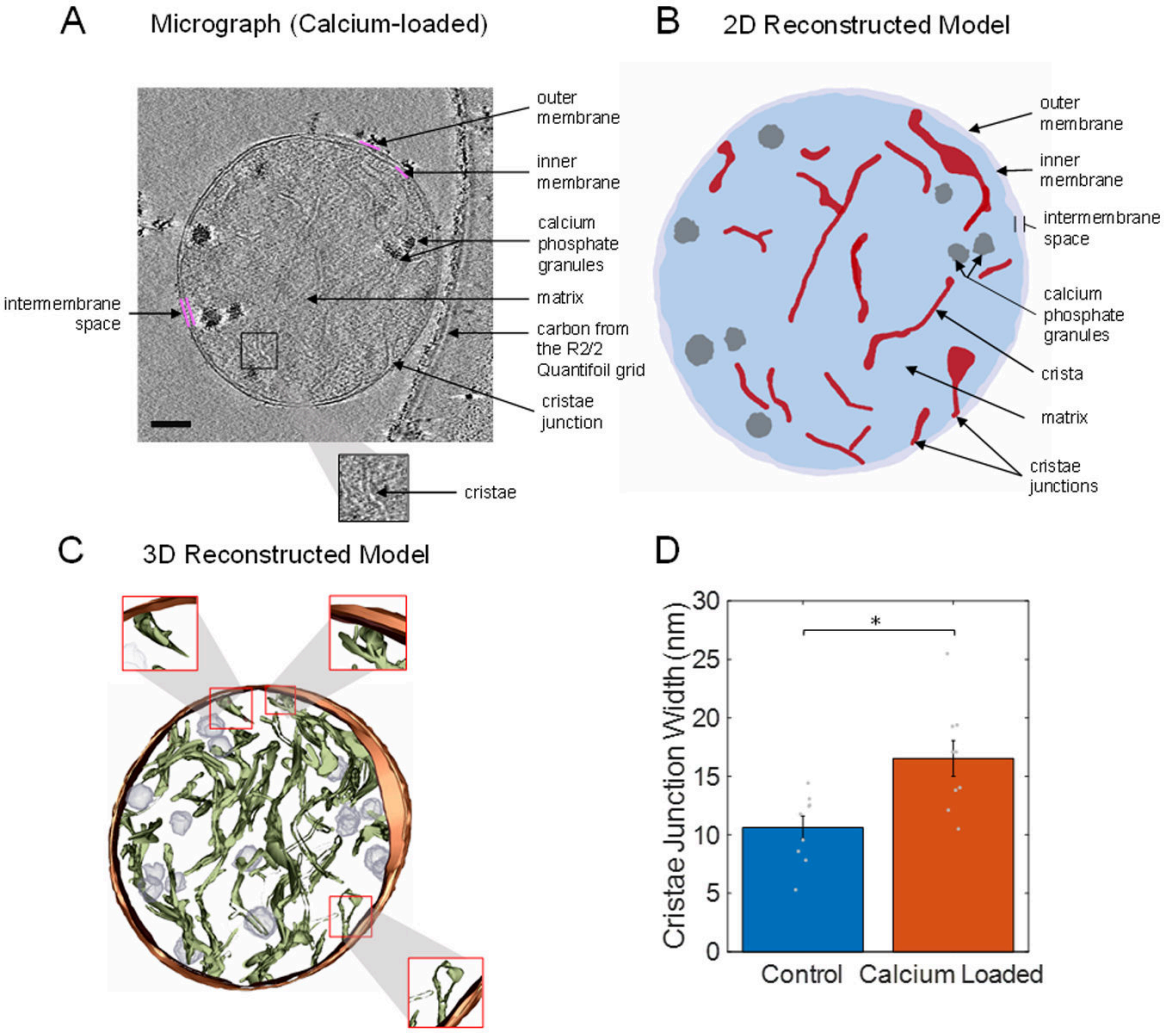
Calcium-loaded mitochondria with granules alters cristae structure. (**A**) Representative portion of a tomogram from a mitochondrion with calcium phosphate granules exposed to a 50 μM bolus of calcium at an approximate resolution of 4 Å. The solid magenta lines highlight the inner or outer membrane boundaries. (**B**) 2D reconstructed model showing that calcium phosphate granules disrupted the cristae membrane surface area, consisting of most of the enzymes responsible for oxidative phosphorylation. (**C**) 3D reconstructed model showing a densely packed, interconnected cristae network. (**D**) Cristae junction widths in calcium-loaded mitochondria, which were significantly larger than the control widths. Approximately 10 cristae junctions from each type of mitochondrion were selected at random and measured. The bars are plotted as means of the data, with error bars representing the standard error of the mean. A * signifies a *p* value less than 0.01. Gray dots are individual data points. Scale bar = 250 nm.

In the absence of calcium, mitochondria possess an intact and dense cristae network, as shown in Figure 2. The cryo-electron micrograph and a ~10 nm thick portion of the corresponding 3D reconstruction show that in the normal state, the internal structure of the cristae network is highly interconnected. In the cryo-EM image, rows of ATP synthase molecules can be seen spiraling down the cristae, with their F1 portion sticking out into the matrix space. The intermembrane and cristae spaces are nearly equal to that of the matrix space. This ensures near-optimal metabolic efficiency by maximizing both the inner membrane surface area and the matrix water space. The inner membrane houses all the proton pumps, transporters, and enzymes required to establish, maintain, and utilize the proton electrochemical gradient for ATP production. The matrix space contains nearly all the enzymes that produce the reducing equivalents used to run the proton pumps. In addition, the cristae junction widths are tight and around 10 nm in diameter (see Figure 3D). The cristae junction serves as a gateway to allow some metabolites, such as ATP, ADP, and Pi into the cristae lumen to be rapidly transported outside the cristae and mitochondria. These junctions also serve to lock key metabolites such as cytochrome c in the cristae lumen. Cytochrome c is a small (~15 kDa) mobile electron carrier that is analogous in function to nicotinamide adenine dinucleotide in the matrix and ubiquinone in the membrane lipid space. By maintaining the cristae network and matrix spaces in the form discussed above, mitochondrial ATP production rates are maximized.

In the calcium-overloaded state, the mitochondrial ultrastructure was significantly altered, as is shown in Figure 3. The presence of calcium phosphate granules in the matrix space had a devastating effect on the intermembrane and cristae spaces. The matrix space was significantly expanded, with the intermembrane and cristae spaces being dramatically reduced. The matrix space expanded due to the increased osmotic pressure in the matrix that occurred when large amounts of calcium were taken up by the mitochondria. In addition, the cristae junctions were significantly wider, being about 17 nm when calcium phosphate granules were present in the matrix (Figure 3D). Based on this finding, we believe that this increase in the cristae junction width led to a redistribution of cytochrome c from the cristae lumen to the intermembrane space. A recent modeling study demonstrated that a small decrease in the cytochrome c concentration in the cristae lumen or an increase in the diffusion distance between complexes III and IV may result in a kinetic disadvantage and lower the maximum electron flux through the respiratory system [51].

This would lead to a decrease in the maximum oxygen consumption rates we observe in calcium-loaded mitochondria. We speculate that the cristae remodeling we saw in our vitrified, isolated mitochondria impaired mitochondrial function by limiting the ability of mitochondria to (1) establish and maintain a large proton electrochemical gradient and (2) impede metabolite diffusion and transport. Additionally, calcium uptake leads to a drop in the membrane potential and an increase in the matrix pH [8,14]. This limits the ability of mitochondria to generate a strong membrane potential and thus lower ATP synthesis rates [4]. In addition, the opening of the cristae junctions leads to a redistribution of cytochrome c from the cristae lumen to the inner membrane space. This further limits the ability of the proton pumps to maintain a local chemiosmotic gradient to support high rates of ATP production.

**Figure 4.**
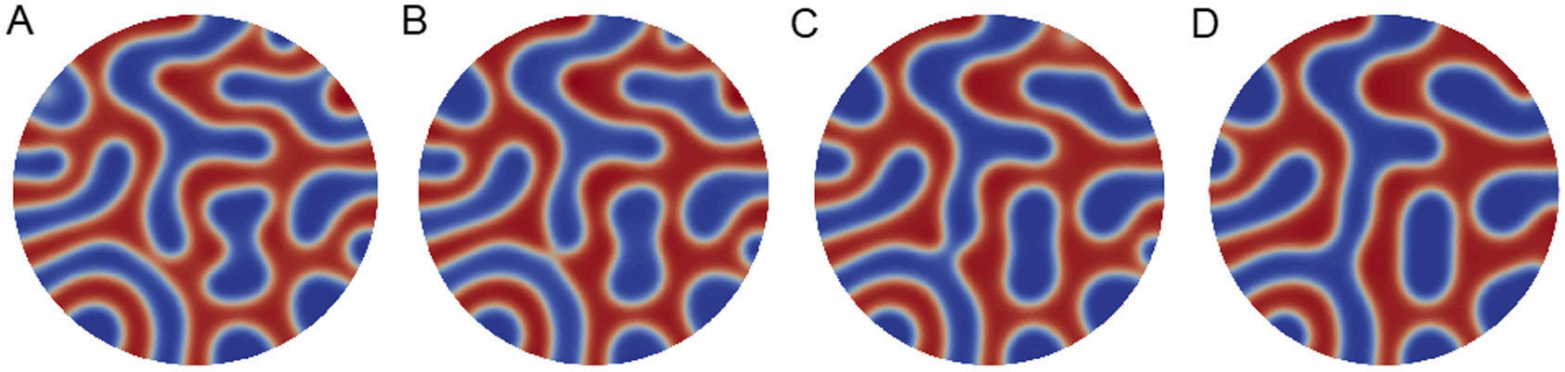
Calcium-free mitochondria simulation by a circle domain with a radius of four. The time step Δ*t* = 0.001 for (**A**) *t*_750_, (**B**) *t*_1250_, (**C**) *t*_1750_, and (**D**) *t*_2250_. Merging and splitting of the matrix and intermembrane spaces are observed as the simulation time is lengthened.

The mitochondrial morphology and the effects of calcium phosphate granules were simulated using the well-established CH model framework. The CH equation was first implemented in the situation of the absence of calcium. In Figure 4, four snapshots of the CH equation simulation are presented with a time step Δ*t* = 0.001 for Equations (14) and (15) on a circle domain with a radius of four (relative to the unit circle). The parameter *γ* is one for the transition region length, which is indicated by the white, rim-like features in Figure 4. The red domain of Figure 4 is the fluid matrix, while the blue domain is the inner membrane. It is shown that in the simulation, the cristae could be observed, and the inner membrane and the fluid matrix could be separated. In the absence of calcium, the inner membrane and the fluid matrix were initially randomly and uniformly distributed in the circle domain.

**Figure 5.**
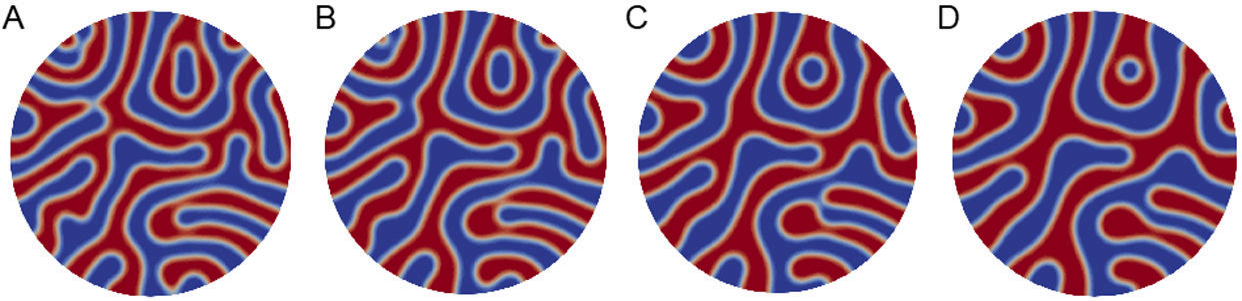
Calcium-free mitochondria simulation by a circle domain with a radius of five. The time step Δ*t* = 0.001 for (**A**) *t*_750_, (**B**) *t*_1250_ (**C**) *t*_1750_, and (**D**) *t*_2250_. As shown in Figure 4, merging and splitting of the phase spaces are observed as the simulation time increases.

In Figure 5, a larger circle domain with radius of five is demonstrated. The parameter *γ* is one for the transition region length, which is indicated by the white, rim-like features in Figure 5. The red domain of Figure 5 is the fluid matrix, while the blue domain is the inner membrane part, which includes the cristae lumen. Compared with Figure 4, thinner cristae can be observed in Figure 5. This was caused by the high-frequency phase interactions introduced by imposing a larger domain. In the comparison with the experimental image, a similar pattern can be observed in Figure 5, in which cristae are randomly and uniformly distributed within the domain.

**Figure 6.**
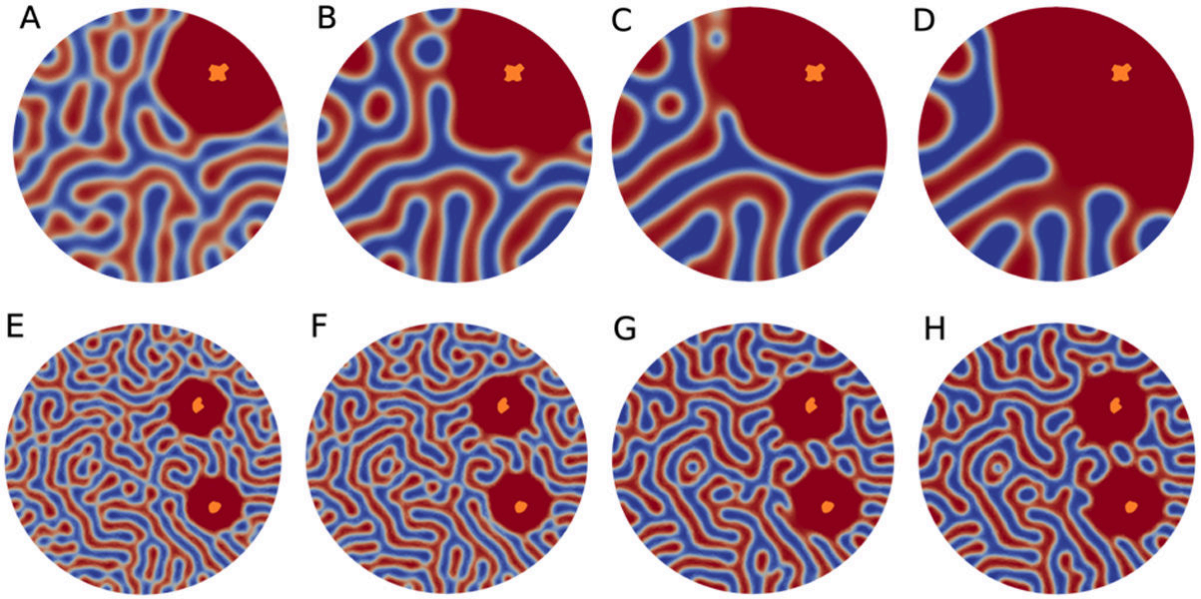
The effect of calcium phosphate granules on the inner membrane morphology. (**A–D**) Simulations on the effect of a single calcium phosphate granule on the mitochondrial cristae network. The Cahn–Hilliard (CH) equation was solved on a circle domain with a radius of five. The object with a white rim is the location of the calcium phosphate granule. The time step Δ*t* = 0.005 for (**A**) *t*_300_, (**B**) *t*_800_, (**C**) *t*_1200_, and (**D**) *t*_1400_. (**E–H**) Two calcium phosphate granule loads of the Cahn–Hilliard equation on a circle domain with a radius of eight. The orange objects are the locations of the loaded calcium phosphate granules. The time step Δ*t* = 0.001 for (**A**) *t*_375_, (**B**) *t*_400_, (**C**) *t*_850_, and (**D**) *t*_900_. As with Figure 3, the matrix and inter membrane spaces can be observed changing dynamically in response to the presence of calcium phosphate granules.

Implementation of the CH model on a mitochondrial structure can help us understand a plausible mechanism that explains the dramatic and titratable decrease in mitochondrial ATP production as a function of calcium overloading. Figure 6 demonstrates the simulations of calcium load cases. In case one, a single calcium phosphate granule was loaded in the first quadrant. In the second case, and additional calcium phosphate granule was loaded in the fourth quadrant. In each panel, the orange objects indicate the location of the calcium phosphate granules. In a fascinating similarity with the experimental observations, the simulations revealed a dramatic reorganization of the cristae membrane because of the presence of calcium phosphate granules. In addition, the matrix space around the granules dramatically expanded. In the simulations, these effects were a result of an energy minimization problem. The presence of these granules near cristae membranes acted as a repulsive force which led to matrix fluid filling the vacated space.

**Figure 7.**
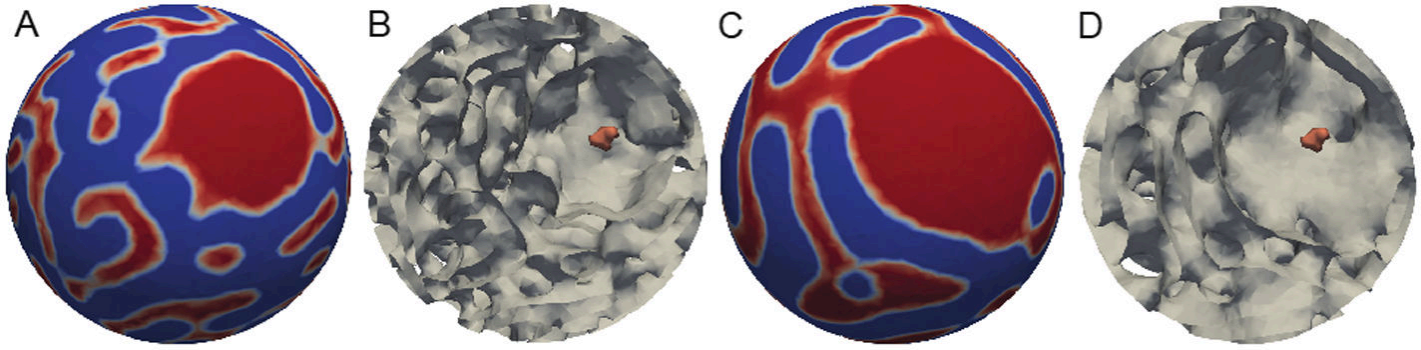
Simulation results on a 3D sphere domain with a radius of five and a single calcium phosphate granule. The time step Δ*t* = 0.001 for (**A**) *t*_500_, (**B**) the inside contour of (**A**), (**C**) *t*_1500_, and (**D**) the inside contour of (**C**). The orange object represents the calcium phosphate granule.

Figure 7 shows the extension of the 2D simulations of the effects of calcium phosphate granules on the inner membrane topology to 3D. In this figure, colored surface plots as well as inside contour plots are presented. It can be seen from the inside contour plots in Figure 7B,D that the granule’s location was at the center of the concave down domain. As the time increased from *t*_500_ to *t*_1500_, the concave down domain enlarged as a direct result of the presence of the calcium phosphate granule.

**Figure 8.**
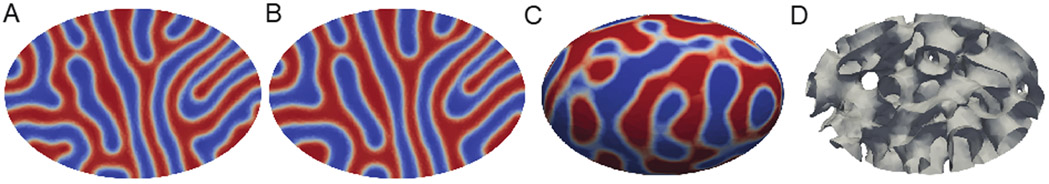
Calcium-free mitochondrial ultrastructure simulation for 2D ellipse and 3D ellipsoid domains. The time step Δ*t* = 0.005 for (**A**) *t*_800_, (**B**) *t*_1000_, (**C**) *t*_60_, and (**D**) the inside contour of (**C**). The boundary domain did not strongly regulate the inner membrane topology.

Changing the geometric boundary conditions from circular and spheroid to elliptical and ellipsoid resulted in a similar inner membrane topology. These results are shown in Figure 8. Simulations of a 2D elliptical mitochondrion with a major axis of 10 and minor axis of 8 are given in Figure 8A,B. In Figure 8C,D, the 3D simulation results of an ellipsoid mitochondrion are presented. A similar pattern can be observed to that in the experimental image, which substantiates the use of the CH model and demonstrates that it can be used to characterize the internal ultrastructural features of mitochondrion with more irregular boundary conditions.

**Figure 9.**
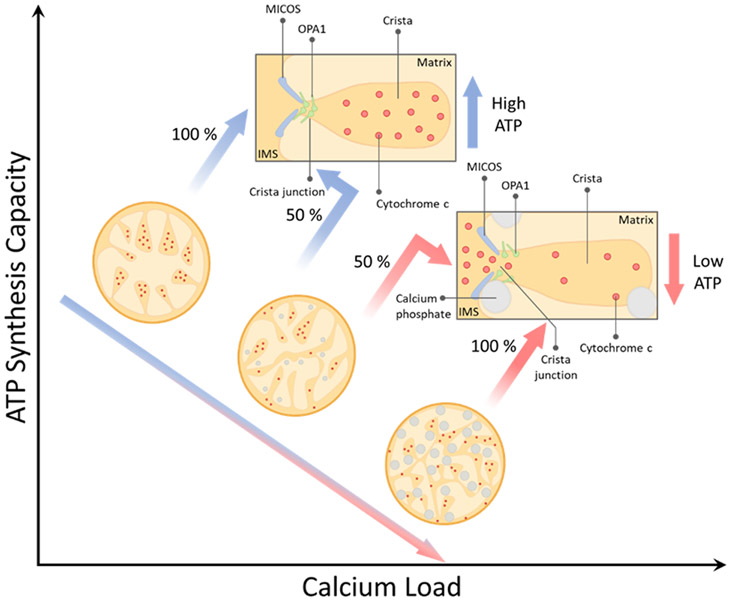
Model of calcium overload-induced mitochondrial dysfunction. We hypothesize that a calcium overload causes cristae remodeling, redistributes cytochrome c, and lowers the ATP synthesis capacity in a manner that is proportional to the calcium load.

From these observations, a preliminary mechanism of calcium-induced mitochondrial dysfunction comes into focus and is presented in Figure 9. The mechanism explains how progressive calcium loading disrupts cristae, the functional units of a mitochondrion, and redistributes key oxidative phosphorylation metabolites. In this model, a calcium overload harms the mitochondrial structure by inducing inner membrane fragmentation and widening cristae junctions, which leads to the inefficient coupling of energy released during substrate oxidation and captured during oxidative phosphorylation.

The mitochondrial morphology is not only affected by calcium phosphate granules [76]. In the 1960s, Hackenbrock showed that mitochondrial ultrastructure configurations lie on a spectrum ranging from the orthodox to the condensed conformation [77]. He later demonstrated that these ultrastructural changes were not associated with the chemical fixation required for electron microscopy [78]. The orthodox conformation is historically defined as the ultrastructural configuration seen under an electron microscope whereby the matrix space is expanded. The inner membrane space reciprocally shrinks in a manner that appears conducive for cristae formation. This conformation is typically observed in the presence of respiratory substrates and the absence of ADP. In the condensed conformation, the matrix is characterized as having a dense or compacted matrix and a reciprocally expanded intermembrane space. This conformation is observed in the presence of ADP or respiratory poisons. Hackenbrock originally proposed that these conformations were explicitly part of a mechanochemical ultrastructural transformation model which facilitated mitochondrial ATP production. This idea was borne out in an era before Peter Mitchell’s chemiosmosis principle was widely accepted [79]. That said, Hackenbrock’s idea was not far off. Configurational changes to the mitochondrial membrane and enzyme localization may indeed be linked with more efficient energy transduction [80]. A common underlying principle coming out of Hackenbrock’s studies reveals that the mitochondrial membrane potential appears to be necessary, but it is not sufficient when it comes to the transition from condensed to orthodox. Therefore, electrostatic effects or volume regulatory elements may be the key to unraveling the ultrastructural changes seen under the electron microscope.

The model presented herein is only the first of many steps being taken to understand the biophysical and biochemical regulatory mechanisms controlling cristae formation and morphology. That said, cristae are specialized compartments of dynamic inner membrane folds whose curvatures are dictated by the dimerization of F_1_F_O_-ATP synthases [37]. In addition to ATP synthase, these specialized microcompartments contain within their membrane the respiratory machinery, or the-so-called electron transfer chain, in addition to energy-linked transhydrogenase, uncoupling proteins, and metabolic carriers. Such a rich environment in ATP synthases not only dictates the cristae shape, but it also supports ATP synthesis by the chemiosmotic coupling resulting from the electrochemical gradient generated by the movement of electrons across the various complexes of the electron transport chain. The sac-like structure shape is stabilized by the mitochondrial contact site and cristae organizing system (MICOS) [81]. This complex is composed of a myriad of proteins, known as Mic proteins, with a variety of roles including membrane shaping, contact site formation, lipid trafficking, protein biogenesis, cristae stability, and scaffolding [81]. While dynamic, the cristae shape has been mostly divided into two main morphotypes: lamellar and tubular. The former is known for its elongated, plate-like three-dimensional appearance, whereas the latter has a blob- or tube-like three-dimensional appearance. Despite the vast knowledge gathered since the discovery of cristae, little is known about how such a dynamic system is tightly regulated or the impact of the multiple morphotypes. It has been hypothesized that the dynamic adaptation of the cristae shape is dependent on the energetic demand, stage of development of the cell, and the cell type [81]. The latter adds a layer of complexity, provided that cells can have predominantly one kind of cristae morphology over the other or a mixture of them [82]. A recent study by Harner et al. showed that while these morphotypes are stabilized by dimers of F_1_F_O_ ATP-synthase and MICOS complexes [83], a lamellar—but not tubular—cristae morphology involves the optic atrophy factor 1 (OPA1), which is known for driving inner membrane fusion events and establishing the cristae junctions [84], whereas studies conducted by Gottschalk et al. using super-resolution structured illumination microscopy identified mitochondrial calcium uptake 1 (MICU1), a protein involved in controlling the activity of the mitochondrial calcium uniporter complex, as a calcium-dependent regulator of the cristae junction [84]. These studies revealed that knocking down MICU1 caused widening of the cristae junctions, membrane depolarization, and higher levels of matrix-resting calcium, similar to the results obtained upon OPA1 knockdown. Hence, while calcium exerts some control over cellular bioenergetics and metabolism [25], these studies revealed a role for calcium in cristae dynamics.

While the modeling approach described herein faithfully reproduced the inner membrane morphology in the normal and calcium-overloaded states, it did have some limitations. For example, we did not include the possible effect that free calcium (calcium ions in the solution) may have had on the proteins that regulated the cristae structure. While we do not yet know of any direct binding of calcium to the putative cristae regulators (e.g., OPA1 and MICOS), there are published studies that indirectly link calcium ions to cristae remodeling [84-88]. We intend to explore this in future studies, identifying the relationship between the total calcium content, ultrastructural changes, and ATP production rates. In this study, the total mitochondrial calcium content was not varied in the cryo-EM studies. If done, we would expect to see the total number and size of the granules play a direct role in the maximal rate of ATP production, as described in Figure 9. In addition, it was not coupled to reaction–diffusion equations to simulate the effects of the ultrastructure on metabolic fluxes, and the location and ultrastructural shaping effects of F_1_F_O_ ATP synthase and other mitochondrial enzymes were not accounted for. An additional limitation of the current model was the absence electrostatic effects on the membrane structure and curvature. Lastly, the model was compared to cryo-EM structural data of isolated mitochondria. It is important to note that the mitochondrial ultrastructural details presented herein may differ substantially from those seen in situ. However, we are exploring how geometric boundary constraints impact cristae morphology and intend to address this situation. In future and ongoing work, each of these limitations will be addressed in order to elucidate their overall impact on the mitochondrial ultrastructure and function.

In conclusion, we presented a novel, phase separation-based approach to model the intermembrane and matrix spaces of mitochondria. These simulation results elucidate a plausible and likely mechanism by which mitochondrial ATP production is compromised at high calcium loads. Future studies will extend this work in order to simulate the volumetric changes and redistribution of key metabolites to test the hypothesis that cristae remodeling caused by calcium loading is the cause of the experimentally observed decrease in the maximum ATP synthesis rates.

## Data Availability

The data and model presented in this study are available from the corresponding author upon request.
